# Risedronate inhibits human osteosarcoma cell invasion

**DOI:** 10.1186/1756-9966-28-105

**Published:** 2009-07-22

**Authors:** Zeng Feng Xin, Yang Kyung Kim, Sung Taek Jung

**Affiliations:** 1 Department of Orthopedic Surgery, Chonnam National University Medical School, Gwangju 501-190, Korea

## Abstract

**Background:**

Osteosarcoma is a highly malignant bone tumor and is the most commonly encountered malignant bone tumor in children and adolescents. Furthermore, significant numbers of patients eventually develop pulmonary metastases and succumb to the disease even after conventional multi-agent chemotherapy and surgical excision. Several solid tumors display enhanced expression of matrix metalloproteinases (MMPs), and recently clinical trials have been initiated on MMP-inhibitors. On the other hand, bisphosphonates (BPs), which have a profound effect on bone resorption, are widely used to treat osteoclast-mediated bone diseases. BPs are also known to inhibit tumor growths and metastases in some tumors such as breast cancer, renal cell carcinoma, and prostate cancer.

**Methods:**

Two osteosarcoma cell lines (SaOS-2 and U2OS) were treated with risedronate (0, 0.1, 1, 10 μM) for 48 hours. Cell viabilities were determined using MTT assay, the mRNA levels of MMP-2 and MMP-9 were analyzed by reverse-transcription polymerase chain reaction, the amount of MMP-2 and MMP-9 protein were analyzed by Westernblot, the activities of MMP-2 and MMP-9 were observed by Gelatin zymography, and Matrigel invasion assays were used to investigate the invasive potential of osteosarcoma cell lines before and after risedronate treatment.

**Results:**

The invasiveness of osteosarcoma cell lines (SaOS-2, U2OS) were reduced in a dose dependent manner follow 48 hour treatment of up to 10 μM of the risedronate at which concentration no cytotoxicity occurred. Furthermore, the gelatinolytic activities and protein and mRNA levels of MMP-2 and MMP-9 were also suppressed by increasing risedronate concentrations.

**Conclusion:**

Given that MMP-2 and MMP-9 are instrumental in tumor cell invasion, our results suggest the risedronate could reduce osteosarcoma cell invasion.

## Background

Osteosarcoma is one of the most common primary malignant tumors of bone and occurs mainly in adolescents and young adults [1,2]. Recently, the prognosis of these patients has improved substantially due to the development of various adjuvant chemotherapies. However, these chemotherapies are not fully effective, and as a result, 20% of all osteosarcoma patients still die owing to tumors metastasis [3-5]. Despite the advances made at improving survival over the last three decades, a limit appears to have been reached [6]. As a consequence, many novel therapies for osteosarcoma are being investigated.

The matrix metalloproteinases (MMPs) are a family of zinc-dependent endopeptidases that remodel and degrade extracellular matrix (ECM). More than 25 MMPs have been identified to date, and are classified based on their substrate specificities and structural characteristics [7-9]. Furthermore, MMPs are considered to play important roles in the matrix degradation for tumor growth, invasion, and tumor-induced angiogenesis [10,11].

Tumor growth, invasion, and metastasis require tumor cell proliferation, proteolytic digestion of the extracellular matrix (ECM), cell migration through basement membranes into the circulatory system, and extravasation and growth at metastatic sites [12]. MMPs contribute to this metastatic process by degrading basement membrane. In addition, MMPs can, due to their proteolytic activities, promote tumor growth by increasing the bioavailabilities of growth factors in the ECM [11]. Furthermore, it is becoming increasingly clear that MMPs play a central role in ECM degradation [13]. Among MMPs, MMP-2 (gelatinase A) and MMP-9 (gelatinase B), are present in large quantities in cancer tissues [14,15], and accumulating evidence indicates that MMP-2 and MMP-9 play critical role during tumor invasion and metastasis [14,16-20]. Furthermore, Matrix metalloproteinases (MMPs) and their endogenous inhibitors participate in the invasive process of human osteosarcoma [21].

Bisphosphonates (BPs) are stable analogues of pyrophosphonate, and are potent inhibitors of osteoclast-mediated bone resorption. They are widely used to treat metabolic bone diseases, such as, Paget's disease [22] and hypercalcemia [23] and to treat postmenopausal osteoporosis [24]. Recently, it was reported that BPs may significantly help control the pain associated with bone tumors [25]. Preclinical evidence suggest that BPs have direct antitumor effects on a variety of human cancer cells [26], and it is known that they decrease cell proliferation in human osteosarcoma cell line panels, disturb the cell cycle, and induce the apoptosis of SaOS-2 cells [27,28]. These findings suggest that BPs could play a beneficial adjuvant role in the treatment of osteosarcoma. However, the inhibitory effects of BPs on osteosarcoma cell have not been comprehensively studied, and therefore, in the present study, we examined the effects of the third-generation BPs, risedronate, on osteosarcoma cell invasion.

## Methods

### Reagents

Risedronate [1-hydroxy-2-(3-pyridinyl)ethylidene]bis [phosphonic acid] was purchased from (Sanofi-Aventis, Korea). A stock solution of risedronate was prepared in phosphate-buffer saline (PBS). All other chemicals and reagents used were of analytical grade.

### Cell Culture

SaOS-2 and U2OS were purchased from the Korean Cell Line Bank (KCLB). Cells were cultivated in Dulbecco's Minimum Essential Medium (DMEM) supplemented with 10% heat-inactivated fetal bovine serum (Gibco BRL, Grand Island, NY). Cultures were maintained at 37°C in a 5% CO_2_/95% air atmosphere. The medium was changed every 2–3 days, and cells were passaged twice a week.

### Risedronate treatment of SaOS-2 and U2OS cells

SaOS-2 and U2OS cells were seeded in 6-well plates at a density of 2 × 10^5^cells/well in DMEM/10% FBS overnight. The cells were then washed and treated with different concentrations of risedronate (0, 0.1, 1, 10 μM) for 48-h at 37°C in 5% CO_2_. Conditioned media were then collected and cells were harvested.

### MTT cell viability assay

SaOS-2 and U2OS cells were seeded onto a 96-well culture plate at a density of 1 × 10^4 ^cells/well in 100 μl of complete DMEM. On the second day of culture, media were replaced with 100 μl of serum-free DMEM and risedronate at concentrations of 0–10 μM. On the third day, 100 μl of 3-(4, 5-dimethylthiazol-2-yl)-2, 5-diphenyltetrazolium bromide (MTT; Sigma, USA) was added to each well and incubated for 4 h. Media were then discarded and 100 μl of dimethyl sulfoxide (DMSO; Sigma) was added. Absorbance was measured at 570 nm using an ELISA reader.

### In vitro invasion

SaOS-2 and U2OS cells (4 × 10^4^) in 300 μl of serum free-MEM were seeded into the upper chamber of a 10-well chemotaxis chamber (Neuro Probe, USA) and complete MEM was placed in the lower chamber, and a Matrigel-coated membrane was inserted between the two chambers. Following overnight incubation at 37°C, the medium in the upper chamber was replaced with serum-free MEM and cells were treated with risedronate at 0, 0.1, 1 and 10 μM for 48 hours incubation at 37°C in a 5% CO_2 _atmosphere. The synthetic MMPs inhibitor, Marimastat (50 μg/mg) was also added to the upper chamber to examine the effect of MMPs on in vitro invasion. The applied concentration of Marimastat was not toxic to the osteosarcoma cells (data not shown). Finally, membranes were fixed and stained using a Hemacolor rapid staining kit (Merck, Germany), and the cells from 5 random microscopic fields (200 × magnification) were counted.

### Gelatin zymography

Protein concentrations in conditioned media were determined using the bicinchonic acid method (BCA kit) (Pierce, IL, USA). Conditioned media was mixed with a equal volume of 4× sample buffer (200 mM Tris-HCl, 8% SDS, 0.4% bromophenol blue, 40% glycerol), and electrophoresed on 8% SDS polyacrylamide gels containing 2 mg/ml of gelatin (type A, Sigma, St. Louis, MO, USA). Gels were then washed twice for 30 min in 2.5% Triton X-100 at room temperature, and incubated for 18 hours at 37°C in incubation buffer (50 mM Tris-HCl (pH 7.5), 5 mM CaCl2, and 200 mM NaCl). Gels were then stained for 1 hour with 0.25% (w/v) Coomassie brilliant blue R-250 (Bio-Rad) and then destained in destaining buffer (10% acetic acid and 20% methanol).

### Western blot analysis

Cells were treated with risedronate (0, 0.1, 1, 10 μM) for 48 h, scraped into 1× cell lysis buffer (Cell Signaling, USA), and incubated for 10 min on ice. The resulting cell lysates were cleared by centrifugation at 6,700 × g at 4°C for 5 min. Supernatants, which contained cytosolic proteins, were collected and protein concentrations were measured using the bicinchonic acid method (BCA kit) (Pierce, IL, USA). Cell lysates, containing same amounts of protein, were mixed with equal volumes of 4× sample loading buffer, boiled for 5 min, cooled on ice for 5 min, and then analyzed by 10% SDS polyacrylamide gel electrophoresis (SDS-PAGE). Separated proteins were transferred to a nitrocellulose membrane (Amersham Life Science, UK), and then the membrane was blocked with 5% skimmed milk in 1× TBST [0.01 M Tris (pH 7.6), 0.1 M NaCl and 0.1% Tween-20] for 1 h at room temperature with shaking and incubated with indicated primary antibodies followed by HRP-conjugated secondary antibody. The immunoreactive protein bands were developed using the Enhanced Chemiluminescence (ECL Plus) system (Amersham Bioscience, UK).

### Reverse transcription-polymerase chain reaction

Cells treated with risedronate (0, 0.1, 1, 10 μM) for 48 h and washed with ice-cold 1× phosphate buffered saline (PBS) twice. Total RNA was extracted using TRIzol Reagent (Invitrogen, USA), according to the manufacturer's instructions. RNA (1 μg) was reverse-transcribed using the Superscript™ First-Strand Synthesis System for RT-PCR (Invitrogen, San Diego) at 37°C. The following primers were used to determine target gene levels. β-actin (sense 5'-CTGGAGCATGCCCGTATTTA-3' and anti-sense 5'-TTTGGTCTTGCCACTTTTCC-3'), MMP-2 (sense 5'-CTCAGATCCGTGGTGAGATCT-3' and anti-sense 5'-CTTTGGTTCTCCAGCTTCAGG-3') and MMP-9 (sense 5'-AAGTGGCACCACCACAACAT-3' and anti-sense 5'-TTTCCCATCAGCATTGCCGT-3'). All primers were checked against the GeneBank Database to ensure no cross-reactivity with other known human DNA sequences. PCR cycles were performed using the following sequence: 94°C for 5 min, then 30 cycles of denaturation at 94°C for 1 minute, annealing at 60°C (for MMP-2) or 58°C (for MMP-9) for 1 minute, and polymerization at 72°C for 1 minute), and followed by 72°C for 7 minutes. RT-PCR products were visualized on 1.2% agarose gels electrophoresed in 0.5 TAE buffer containing 0.5 μg/ml ethidium bromide.

### Statistical analysis

Band Intensities were quantified using Multi Gauge V3.0 and Scion Image software. Results are expressed as means ± standard deviations. Statistical significance was accepted for p values of < 0.05 by the Kruskal-Wallis Test and Mann-Whitney *U *test, and all statistical analyses were reviewed independently by a statistician.

## Results

### The antiproliferative effects of risedronate on SaOS-2 and U2OS cells

**Figure 1:**
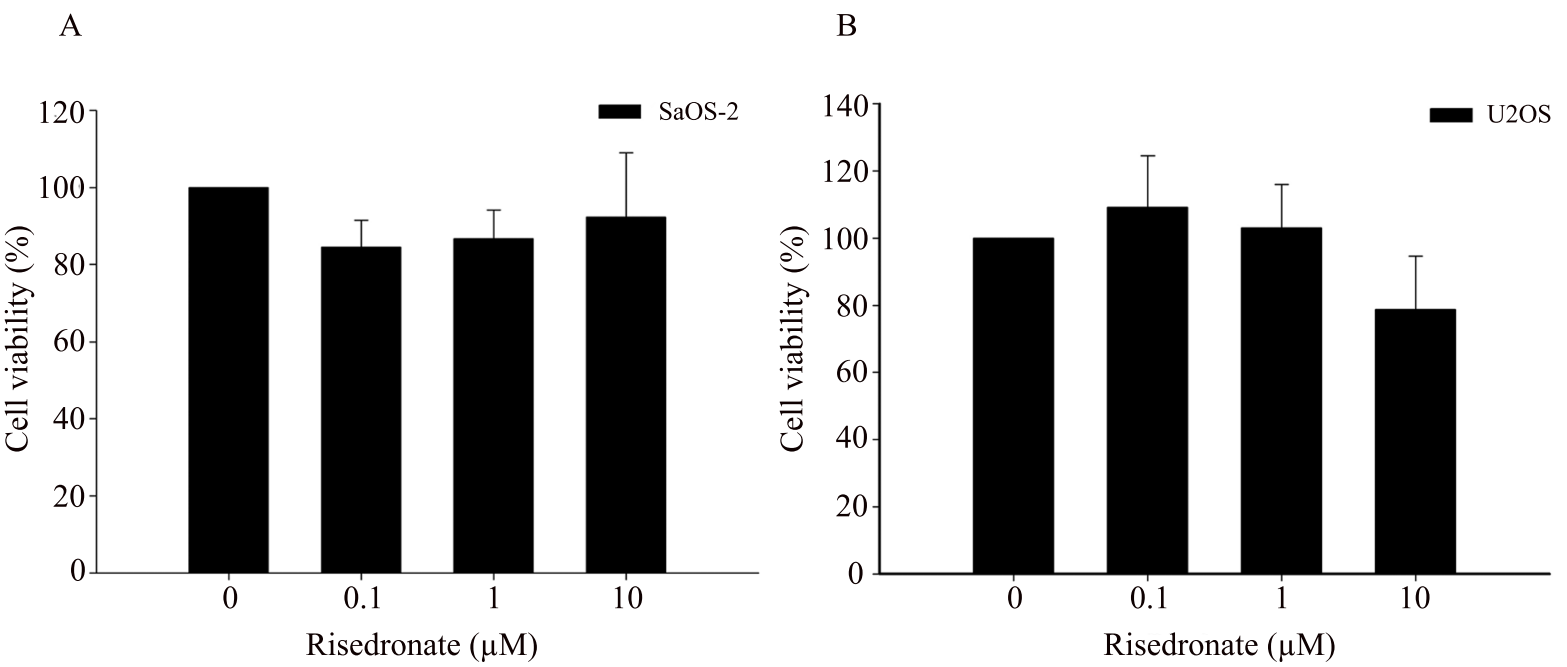
**Risedronate at concentrations up to 10 μM had no cytotoxic effect on either SaOS-2 or U2OS cells**. Both cell lines in serum-free MEM were treated or not with the indicated concentrations of risedronate and then incubated for 48 h before doing MTT assay for cell growth quantification. The bar graph shows the absorbance (expressed as percentages of controls) measured at 570 nm on an ELISA reader (n = 3 independent experiments; mean ± standard deviation is shown).

MTT assays were used to determine the effects of risedronate on osteosarcoma cell growth. Risedronate treatment at 0 to 10 μM for 48-hours did not significantly inhibit the growth of either cell-line (Fig. 1), demonstrating that it has no significant effect on SaOS-2 or U2OS survival at a concentration of 10 μM. Thus, we performed all subsequent experiments using risedronate concentrations between 0 and 10 μM

### Risedronate suppressed the invasive capacities of SaOS-2 and U2OS cells

**Figure 2:**
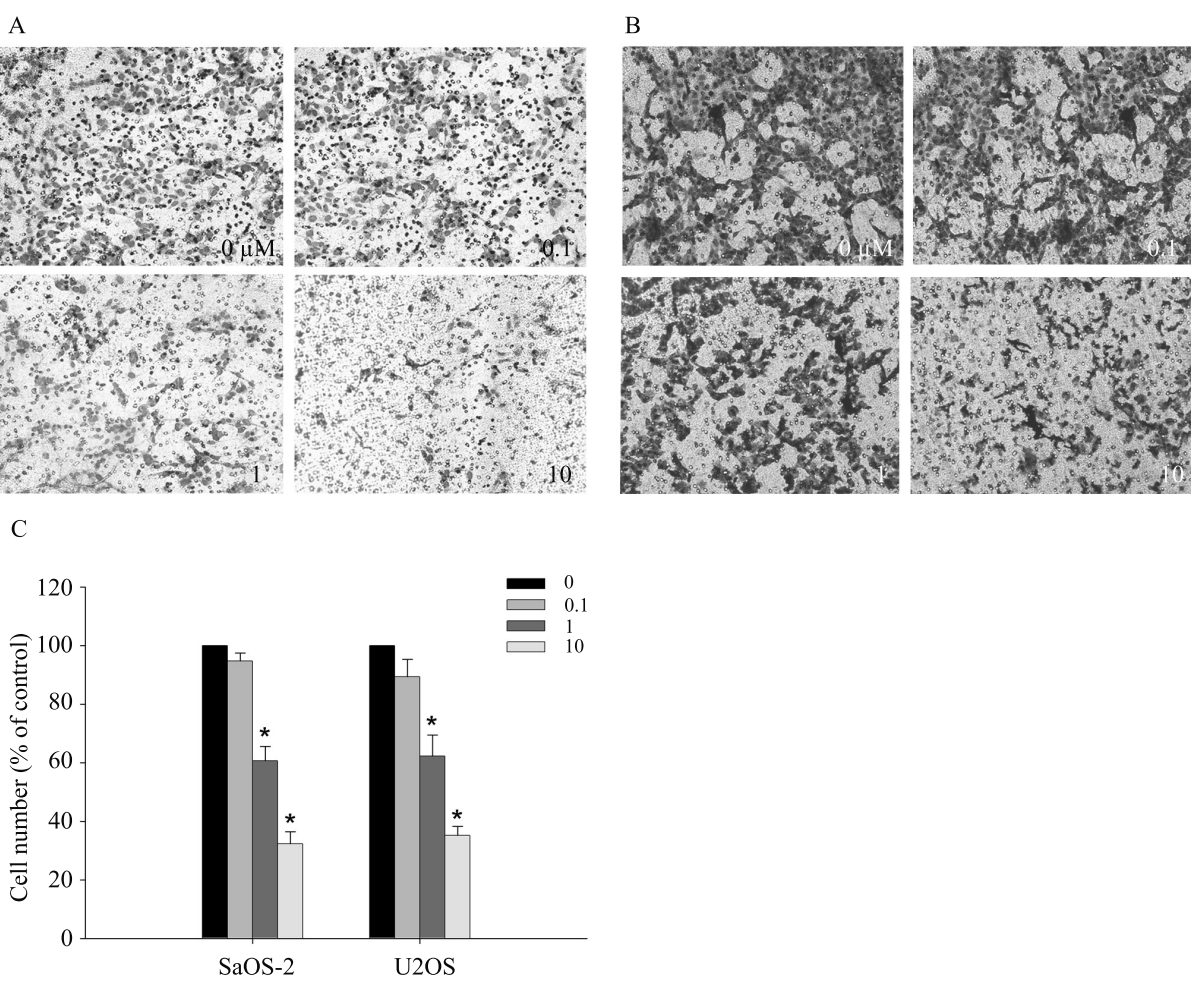
**Risedronate impedes the invasiveness of SaOS-2 and U2OS cells (A and B)**. A 10-well chemotaxis chamber was used to measure the effect of risedronate on invasiveness. A Matrigel-coated membrane was inserted between the upper and lower chambers, and stained using a Hemacolor rapid staining kit. Stained areas represented numbers of migrating cells. The numbers in the panels show the concentration of risedronate added. Images are representative of three independent experiments. Bars (C) represent cells number (expressed as percentages of controls) of each image ± standard deviation.

**Figure 3:**
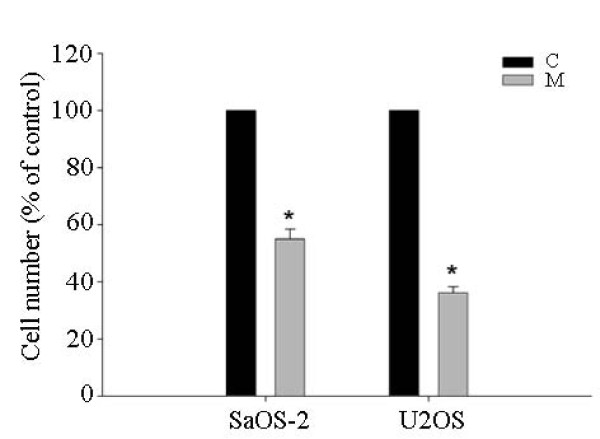
**MMP-inhibitor Marimastat (50 μg/mg) impedes the invasiveness of SaOS-2 and U2OS cells**. Three different experiments with each cell line were performed. Bars represent the cell numbers (expressed as percentages of controls) of each image ± standard deviation. Abbreviations: C: control; M: Marimastat.

We carried out Matrigel invasion assays after treating SaOS-2 and U2OS cells with risedronate. Risedronate was found to inhibit the invasive activities of both cell lines dose-dependently (p < 0.05) (Fig. 2). We also examined the MMP-inhibitor marimastat in the invasion assay to investigate a possible relationship between invasion capacity and MMP expression. Marimastat inhibit the both cells invasion significantly (SaOS-2: 55 ± 6%, U2OS: 36 ± 4%, p < 0.05) (Fig. 3).

### Risedronate reduced MMP-2 and MMP-9 activities in SaOS-2 and U2OS cells

**Figure 4:**
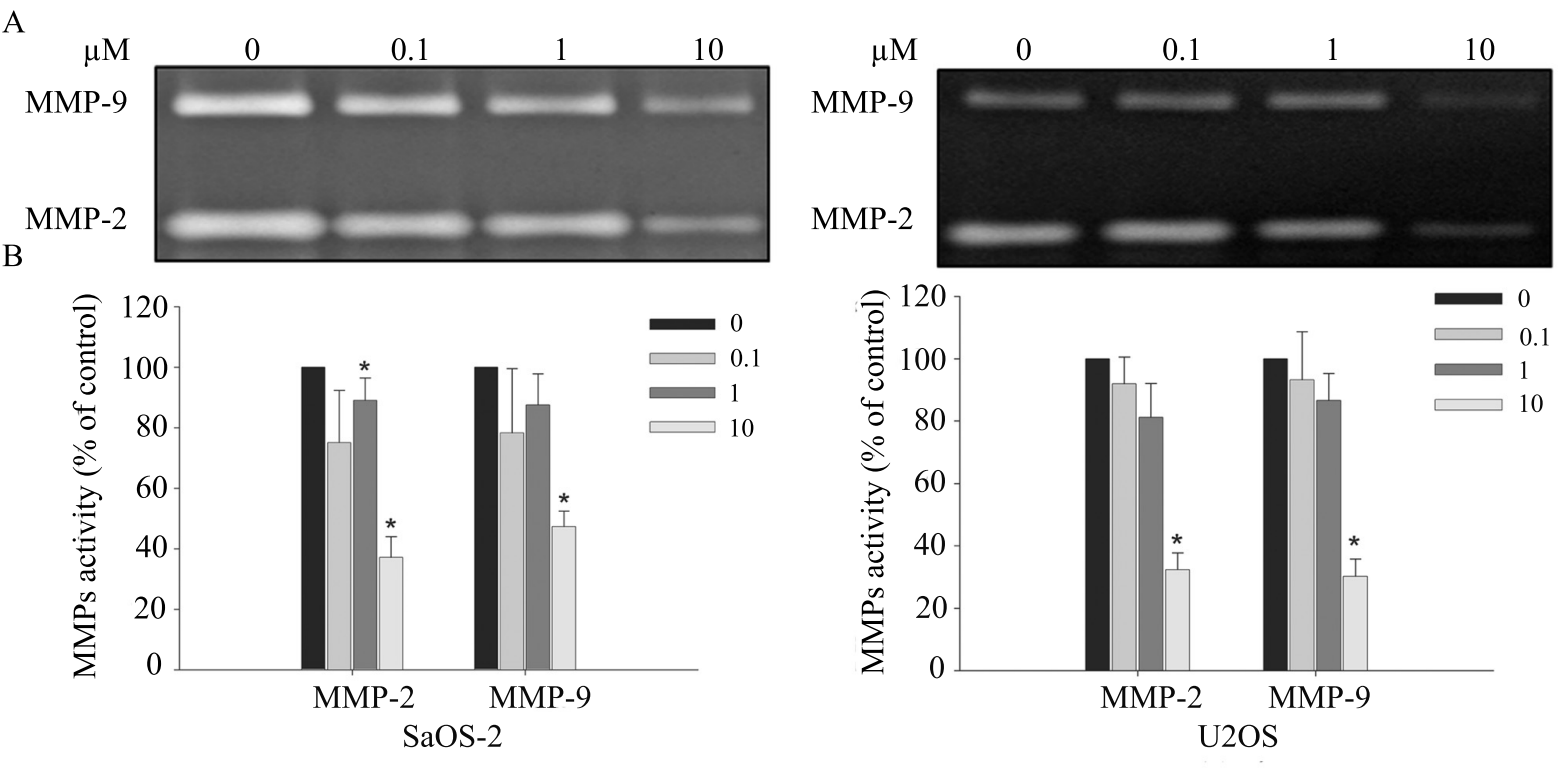
**Risedronate inhibited the gelatinolytic activities of MMP-2 and MMP-9**. (A) Conditioned media harvested from SaOS-2 and U2OS cells treated for 48 h with the indicated concentrations of risedronate were analyzed by gelatin zymography. The white bands represent MMP-mediated gelatin digestion. The image is representative of three independent experiments. MMPs activities (expressed as percentages of controls) are shown in B (n = 3). Numbers in boxes represent the concentration of risedronate (in μM) added to cells. Bars represent the MMPs activities (expressed as percentages of controls) of each band ± standard deviation.

Since MMP-2 and MMP-9 play a critical role in tumor cell invasiveness, we examined the effect of risedronate on the enzyme activities of MMP-2 and MMP-9. Accordingly, gelatin zymography was conducted using conditioned media harvested from risedronate treated SaOS-2 and U2OS cells. The gelatinolytic activities of both MMP-2 and MMP-9 were found to be reduced in both cell lines after treatment with increasing concentrations of risedronate, which suggested that the reductions in cell invasion by risedronate is a consequence of reductions in the activities of MMP-2 and MMP-9 (p < 0.05) (Fig. 4).

### Risedronate reduced MMP-2 and MMP-9 protein levels in both cell lines

**Figure 5:**
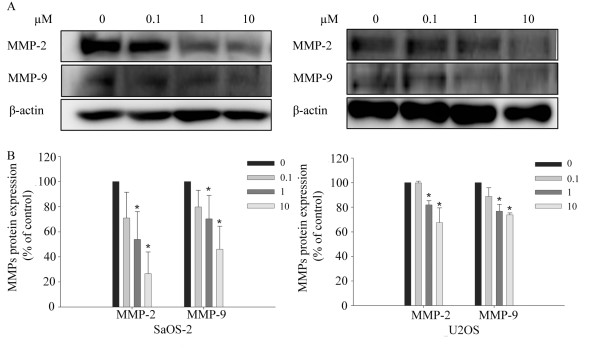
**Risedronate reduced the expressions of MMP-2 and MMP-9 proteins in SaOS-2 and U2OS cells**. (A) Cells were treated with the indicated concentrations of risedronate for 48 h, and then cell lysates were Western blotted. Beta-actin was used as a loading control. Images are representative of three independent experiments. B shows MMPs protein levels (expressed as percentages of controls) (n = 3). Numbers in the box represent the concentration of risedronate in μM added to the cells. Bars represent MMPs protein levels (expressed as percentages of controls) of each band ± standard deviation.

To investigate whether risedronate inhibits the expressions of MMP-2 and MMP-9, SaOS-2 and U2OS cells were treated with risedronate and MMP-2 and MMP-9 protein levels were determined by Western blotting. As shown in Fig. 5, Western blotting revealed that risedronate inhibit MMP-2 and MMP-9 protein levels (p < 0.05).

### Risedronate suppressed MMP-2 and MMP-9 mRNA levels in both cell lines

**Figure 6:**
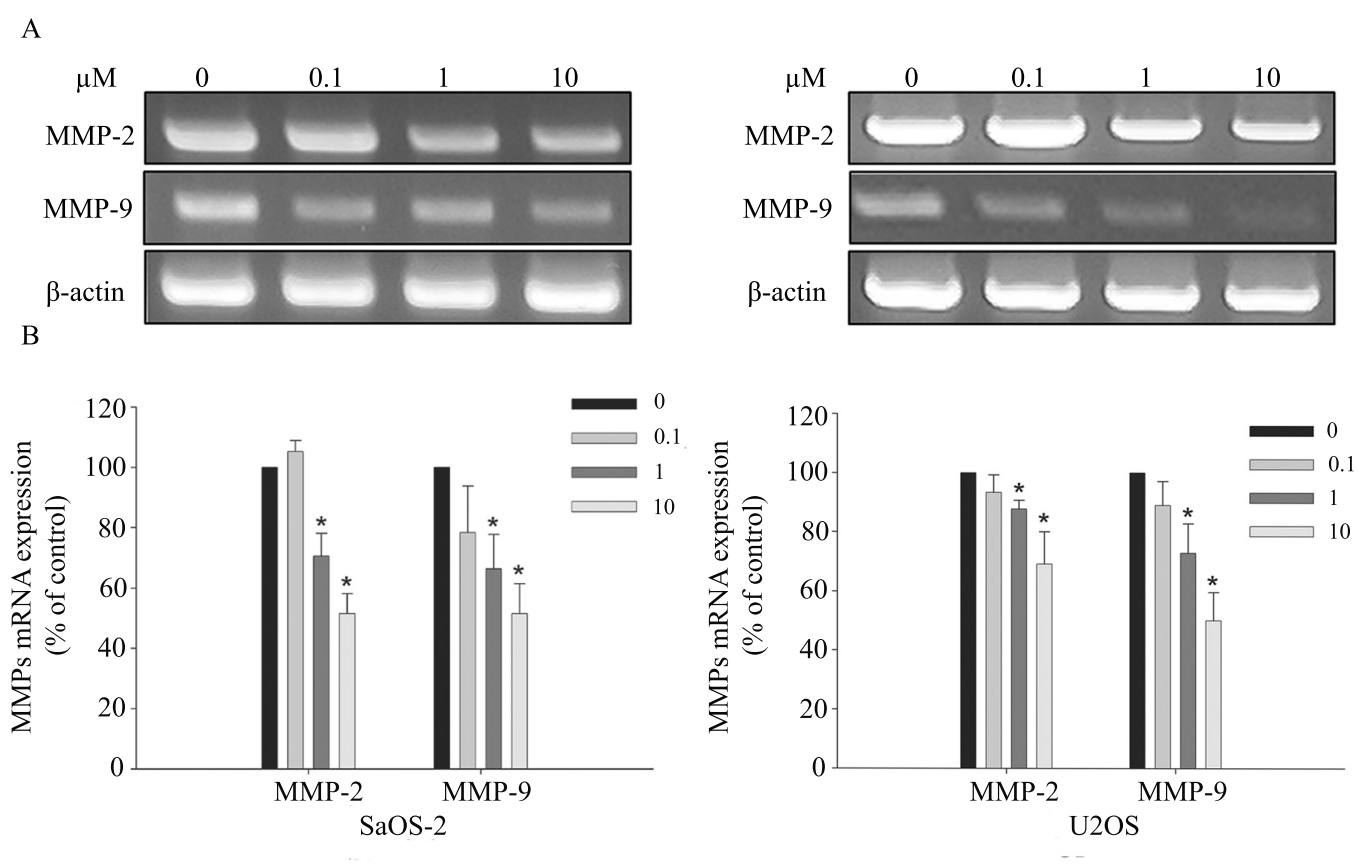
**Risedronate suppressed the expressions of MMP-2 and MMP-9 mRNA in SaOS-2 and U2OS cells**. (A) Cells were treated with the indicated concentrations of risedronate for 48 h and then processed for RT-PCR. Beta-actin was used as a loading control. Images are representative of three independent experiments. MMPs mRNA levers (expressed as percentages of controls) are shown in B (n = 3). Numbers in the box represent the concentration of risedronate in μM added to the cells. Bars represent MMPs mRNA levels (expressed as percentages of controls) of each band ± standard deviation.

RT-PCR was used to determine whether risedronate suppresses MMP-2 and MMP-9 at the transcription levels. Risedronate was found to attenuate MMP-2 and MMP-9 mRNA levels dose-dependent in both cell lines (p < 0.05) (Fig. 6).

## Discussion

Osteosarcoma is an aggressive malignant bone disorder exerting a high potential to invade and metastasize. A number of studies have demonstrated the beneficial effects of bisphosphonates on bone metastases from different solid tumors, such as, those of the breast, prostate and renal cell carcinoma [29,30]. In the majority of previous studies, first or second-generation bisphosphonates have been examined at the relatively high concentrations required to inhibit the cell proliferation of osteosarcoma cells [31,32]. In addition, third-generation bisphosphonates have been reported to induce osteosarcoma cell apoptosis. Evdokiou and colleagues studied the third-generation bisphosphonate, zoledronic acid (ZOL), and found that it dose- and time-dependently decreased cell proliferation in a panel of human osteosarcoma cell lines [27], Tadahiko Kubo and Shoji Shimose reported that minodronate and incadronate perturb the cell cycle and induce the apoptosis of SaOS-2 cells [28]. However, the molecular mechanism underlying inhibition by BPs has not been determined. Cheng YY et al. reported that alendronate reduces MMP-2 secretion and induces tumor cell apoptosis in osteosarcoma [33], but the molecular targets and modes of action of MMP-2 and MMP-9 inhibitors, like risedronate, are substantially unknown. In the present study, we found that risedronate suppresses cell invasion and the gelatinolytic activities and protein and mRNA expressions of MMP-2 and MMP-9 in the SaOS-2 and U2OS osteosarcoma cell lines. Pia Heikkilä, in a study on the inhibition and downregulation of MT1-MMP by clodronate (a non-nitrogen-containing bisphosphonate) suggested that these activities are related to reductions in MG-63 osteosarcoma cell invasion and spread [34]. Pro-MMP-2 can be activated by several mechanisms depending on stimulators and cell types. In particular, pro-MMP-2 can be activated by highly expressed MT1-MMP and adequately expressed TIMP-2 [35,36]. Accordingly, our results indicate that further research is required on the roles played by TIMP-2 and MT1-MMP.

MMP-9 is considered to be particularly good targets for anticancer drugs because it degrades gelatins, which are major components of the basement membrane. The expression of MMP-9 correlated with an aggressive, advanced invasive or metastatic tumor phenotype [37,38]. In the present study, the MMP-inhibitor Marimastat significantly inhibited osteosarcoma cell invasion, which suggest that MMPs are vital factor in osteosarcoma invasion, and that risedronate suppressed the expressions of MMP-2 and MMP-9. Accordingly, our findings demonstrate that risedronate has anti-invasive and antimetastatic activity via the inhibition of MMP-2 and MMP-9 activity in human osteosarcoma cells. On the other hand, Ichinose et al found that bisphosphonates alone do not influence the amount of MMP-2 produced by human osteoblasts, which suggests that bisphosphonates suppress expression of MMPs in osteosarcoma cells but not in normal human osteoblasts [39].

According our MTT assay results, risedronate at up to 10 μM had no significant cytotoxic effect on SaOS-2 or U2OS cells. Therefore, given the known importance of MMP-2 and MMP-9 in tumor invasion, our findings suggest that the inhibitory effect of risedronate on osteosarcoma cell invasion is probably due to MMP inhibition rather than tumor cell death.

## Conclusion

This study suggests that risedronate downregulates the expressions of MMP-2 and MMP-9 in osteosarcoma, and that this is responsible for its effect on osteosarcoma cell invasiveness. This report provides first evidence that risedronate downregulates the expressions and activities of MMP-2 and MMP-9 in osteosarcoma cells in vitro.

## Abbreviations

BPs: bisphosphonates; MMPs: matrix metalloproteinases; RT-PCR: Reverse transcription-polymerase chain reaction.

## Competing interests

The authors declare that they have no competing interests.

## Authors' contributions

In our study, all authors are in agreement with the content of the manuscript. Each author's contribution to the paper: XZF: First author, study design, data analysis, experimental studies, manuscript editing. KYK: study design, experimental studies, data analysis. JST: Corresponding Author, study design, experimental studies, data analysis, manuscript preparation.
